# Extraction and Calculation of Roadway Area from Satellite Images Using Improved Deep Learning Model and Post-Processing

**DOI:** 10.3390/jimaging8050124

**Published:** 2022-04-25

**Authors:** Varun Yerram, Hiroyuki Takeshita, Yuji Iwahori, Yoshitsugu Hayashi, M. K. Bhuyan, Shinji Fukui, Boonserm Kijsirikul, Aili Wang

**Affiliations:** 1 Department of Electronics & Electrical Engineering, Indian Institute of Technology Guwahati, Guwahati 781039, India; mkb@iitg.ac.in; 2 Graduate School of Engineering, Chubu University, Kasugai 487-8501, Japan; htake@isc.chubu.ac.jp (H.T.); iwahori@isc.chubu.ac.jp (Y.I.); y-hayashi@isc.chubu.ac.jp (Y.H.); 3 Department of Information Education, Aichi University of Education, Kariya 448-8542, Japan; sfukui@auecc.aichi-edu.ac.jp; 4 Department of Computer Engineering, Chulalongkorn University, Bangkok 10330, Thailand; boonserm.k@chula.ac.th; 5 Higher Educational Key Laboratory for Measuring and Control Technology and Instrumentations of Heilongjiang, Harbin University of Science and Technology, Harbin 150080, China; aili925@hrbust.edu.cn

**Keywords:** roadway extraction, area calculation, satellite images, U-net++, ResNeXt, Pix2Pix, deep learning model

## Abstract

Roadway area calculation is a novel problem in remote sensing and urban planning. This paper models this problem as a two-step problem, roadway extraction, and area calculation. Roadway extraction from satellite images is a problem that has been tackled many times before. This paper proposes a method using pixel resolution to calculate the area of the roads covered in satellite images. The proposed approach uses novel U-net and Resnet architectures called U-net++ and ResNeXt. The state-of-the-art model is combined with the proposed efficient post-processing approach to improve the overlap with ground truth labels. The performance of the proposed road extraction algorithm is evaluated on the Massachusetts dataset and it is shown that the proposed approach outperforms the existing solutions which use models from the U-net family.

## 1. Introduction

The calculation of the roadway area from openly available satellite images aims to automate the analysis of the total road area covered by satellite images. The task uses open-source satellite images with a specified resolution to calculate the entire road area covered in the image view. We formulate this task as a two-step problem. The first step is the roadway extraction from the given satellite images, while the second step involves calculating the roadway area using pixel resolution.

Existing approaches to solving the first step use deep learning architectures to generate a semantic map of the road line [1,2,3,4,5,6,7,8,9,10,11,12,13], which is then post-processed to obtain a final road mask. These approaches involve using U-net family models [14], which are state-of-the-art architectures for roadway extraction problems.

Zhou et al. [15] proposed an improved variant of U-net, U-net++, which outperformed U-net in medical image segmentation. This paper aims to analyze the performance of this architecture on the roadway extraction problem. We also highlight the significance of using aggregated residual transformations, such as ResNeXt [16], in place of regular residual networks [7]. Semantic models such as U-net [15] generate a noisy pixel map of the roadway mask. The pixel map should be subjected to post-processing to obtain a clean binary mask. In this study, we propose an extensive post-processing method that outperforms the previous methods combined with the deep learning architectures.

We also introduce a simple mathematical formulation to solve the second step of calculating road area from the satellite image. This numerical approach uses pixel ratio and resolution to estimate the final result.

## 2. Materials and Methods

### 2.1. Methodology

**Figure 1:**
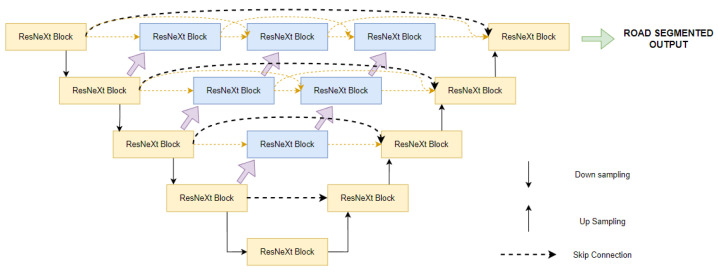
The U-net++ with ResNeXt blocks consist of an encoder and decoder with nested dense convolutional skip connections. The main idea behind U-net++ is to bridge the semantic gap between the feature maps of the encoder and decoder prior to fusion. Here, dark-black lines indicate original U-net, and orange and blue lines indicate U-net++ components introduced by Zhou et al. [15].

This section describes the U-net++ model for roadway extraction in detail. We start by explaining the basic overview of the model followed by the components, U-net++, and ResNeXt block shown in Figure 1. Training strategy, augmentations, and post-processing pipelines are also explained in the later sections.

#### 2.1.1. Model Overview

In order to improve the results of the existing U-net model, Zhou et al. [15] proposed U-net++. The paper introduces a denser U-net model with more skip connections. They hypothesize that features maps should be enriched with information from lower layers before fusing into a decoder network.

Zhou et al. [15] argues that the dense convolutional blocks, whose number of layers depend on the pyramid layer, bring the semantic level of encoder maps closer to that of feature maps waiting in the decoder, and that the optimizer would face an easier optimization problem when the received encoder feature maps and the corresponding decoder feature maps are semantically similar.

Because of skip pathways, U-net++ produces full-resolution feature maps at various semantic levels, allowing us to supervise the model in the deep layers and prune the model.

#### 2.1.2. ResNeXt: Aggregated Residual Blocks

Xie et al. [16] suggested using an aggregated set of residual transformations instead of plain transformations. Cardinality is defined as the size of this set of transformations. Xie et al. argue that increasing this cardinality is a more effective way of gaining accuracy than going deeper or wider. Consider *T*, a transformation consisting of multiple weighted neural layers. This is then aggregated *C* times to form F(x)

(1) $$F\left(x\right)=\sum_{k=1}^{C}T_{k}\left(x\right)$$

**Figure 2:**
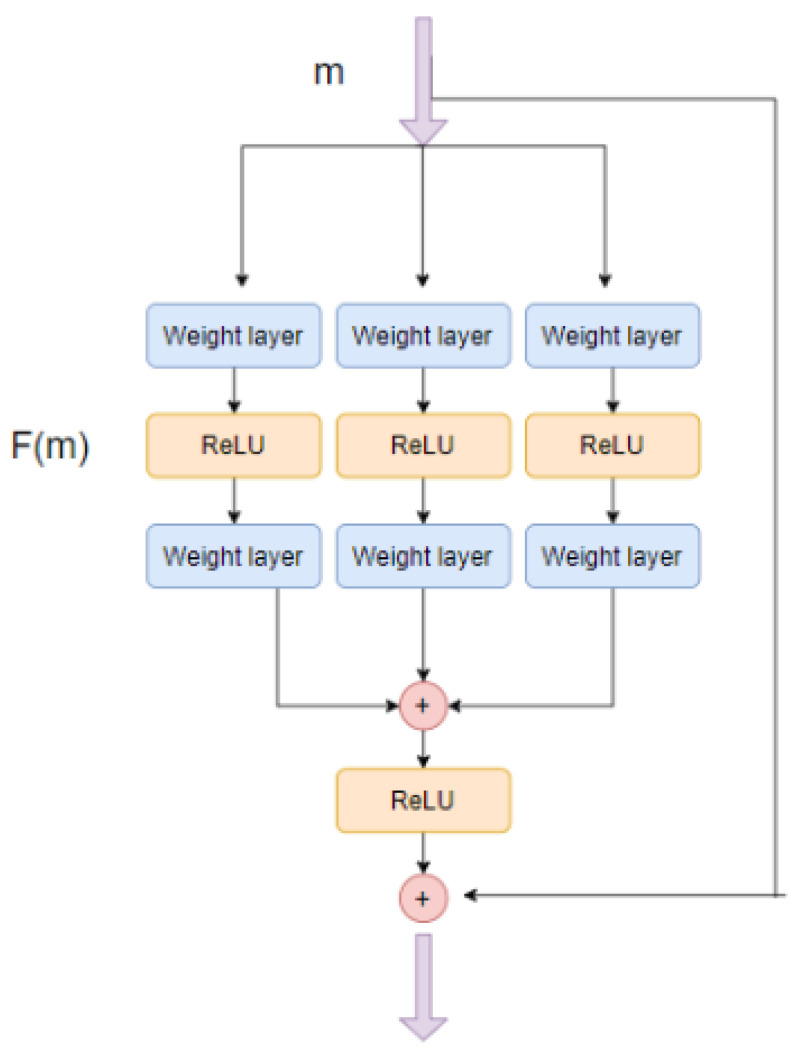
A ResNeXt block with skip connection This block represents Equation (2) with *C* = 3.

The block is then connected residually. The residual connection is also visualized in Figure 2.

(2) $$F\left(x\right)=x+\sum_{k=1}^{C}T_{k}\left(x\right)$$

This mathematical relation can be expressed as a layer diagram, as shown in Figure 2. It is important to note that the topology of all the weight layers on the same level is the same.

The ResNeXt architecture exploits the *split–transform–merge* strategy, along with the usual ResNet’s way of repeating layers. It outperforms ResNet on the ILSVRC 2016 Classification task and ImageNet datasets.

#### 2.1.3. Post-Processing: Result Refinement

The U-net++ with ResNeXt backbone outputs a probability map for the road segmentation output. There is a need to convert it to a binary mask to obtain the extraction output. The primitive way involves setting a threshold to generate the mask directly. The approach presented in Section 2.2.9 describes an extensive refinement pipeline in order to clean up the noise and faulty predictions. The post-processing pipeline is the most important contribution in this paper. The detailed post-processing is explained in the later sections.

### 2.2. Experiments and Metrics

This section describes experiment settings and the metrics that were evaluated via experiments.

#### 2.2.1. Dataset

For testing our algorithm on openly available images, we chose the Pix2Pix Maps dataset, which was introduced by Isola et al. [17]. The Pix2Pix dataset was created to train Pix2Pix GAN Models. We use the aerial-to-map part of Pix2Pix.This paper also uses the Massachusetts Roads dataset to test the proposed model and show that it outperforms the previous models of the U-net type.

#### 2.2.2. Pix2Pix Dataset

**Figure 3:**
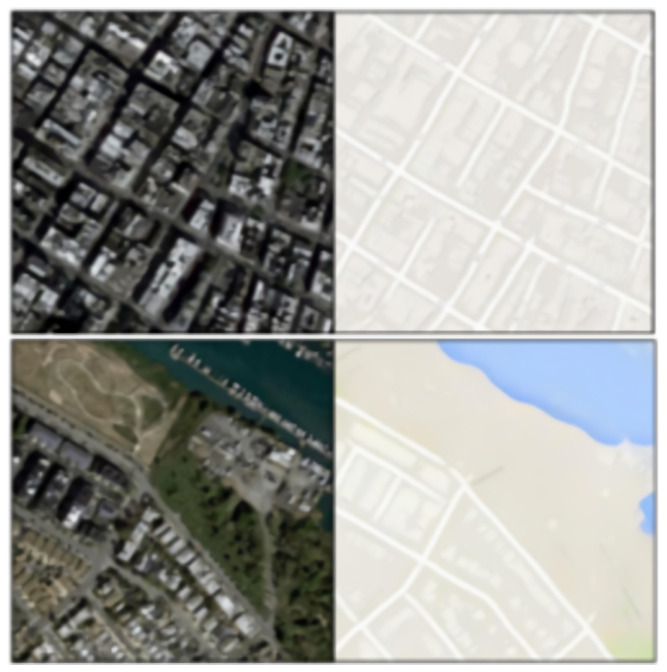
An example of aerial-to-map dataset of Pix2Pix images. These images were used for training and testing our models.

The Pix2Pix dataset consists of various datasets, such as labels to street scenes, labels to the facade, black and white to color, aerial-to-map dataset, day to night, and edges to photo. We focus on the aerial-to-map dataset in this study. An example of an aerial-to-map dataset is shown in Figure 3.

The dataset is divided into training and validation, consisting of 1096 and 1098 images, respectively. These images are scraped from Google Maps images across the Manhattan region, New York, which makes them openly available.

**Figure 4:**
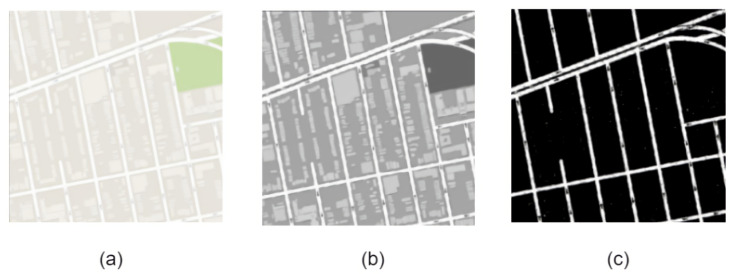
Pre-processing of Pix2Pix images—(**a**) initial image of the map (**b**), converting to black and white (**c**), thresholding to obtain the binary mask.

**Figure 5:**
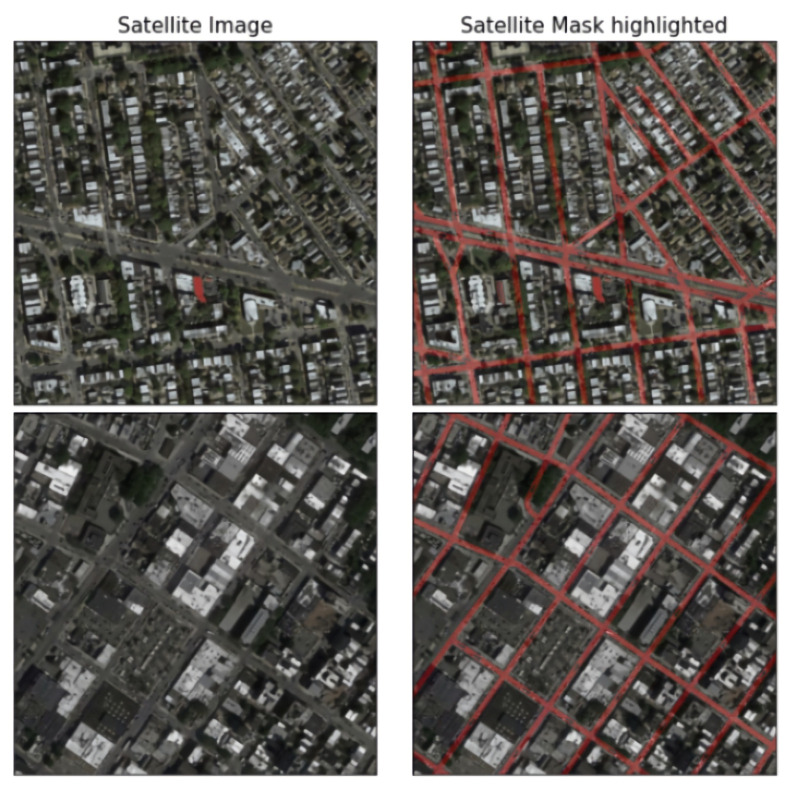
The binary masks are colored red and overlaid onto the satellite image to visualize the roads in the satellite images. This is useful to visualize the outputs.

The proposed U-net++ model takes the input of the satellite image and outputs the binary mask. Therefore, we convert all the map images into binary masks. The image is first converted to grayscale and then thresholded to obtain a binary mask. Various threshold values are explored to obtain the final mask. This process is visualized in Figure 4. These binary masks are overlaid on the original images to visualize the roads in the images, as shown in Figure 5.

#### 2.2.3. Massachusetts Dataset

This paper uses the Massachusetts dataset for testing the roadway extraction model. A lot of previous works on roadway extraction are focused on this dataset [2,3,4,6]. Hence the proposed model is trained on the dataset. The Massachusetts Roads dataset was built by Mihn et al. [18]. The dataset consists of 1171 images in total, including 1108 images for training, 14 images for validation, and 49 images for testing. The size of all the images in this is 1500 × 1500 pixels with a resolution of 1.2 m per pixel.

Massachusetts provides us with binary masks to predict roadways, and we use the same training strategy for this dataset as with Pix2Pix.

#### 2.2.4. Augmentations

Deep neural networks require huge datasets to work with. Large datasets provide generalization capabilities to the models. Both Pix2Pix and Massachusetts datasets have around 1000 images each, and using them directly does not provide generalization capability to our models. We used *on-the-fly* augmentation on the datasets while training the models. *On-the-fly* augmentation involves performing random augmentation to the image before training the model.

This generates a different image every time the dataset is accessed, the augmentation is generated randomly. Although this does not allow us to reproduce the results exactly, it gives us a huge advantage when compared to manually augmenting the dataset and storing it, as it does not use disk space. Training for a large number of epochs with small random augmentations ensures that the model always converges to approximately the same minimum. The following are the augmentations explored in the training:

- **Horizontal Flip**: Flips the image on the horizontal axis;
- **RandomCrop**: Takes small-sized crops from the image. This was especially important in the Massachusetts dataset, as the high resolution does not allow the model to learn fine features. The crops provide a *zooming* effect to the image, which helps the model to learn small-scale features, such as road edges, buildings, etc;
- **Random Brightness**: This changes the brightness of the images. This helps our model to generalize for night and day images;
- **CLAHE**: Contrast-limited adaptive histogram equalization prevents the over-amplification of noise resulting from the AHE;
- **Random Blur**: Blurs the images with random kernel sizes to allow the model to focus on larger features.

#### 2.2.5. Loss and Metrics

This paper uses a combination of Dice loss and cross-entropy to train the model. Dice loss is defined as one minus the Dice coefficient.

(3) $$L_{\mathrm{BCE}}=-\frac{1}{N}\sum_{n=1}^{N}\left(\bar{y_{n}}log\left(y_{n}\right)+\left(1-\bar{y_{n}}\right)log\left(1-y_{n}\right)\right)$$

(4) $$L_{\mathrm{DICE}}=1-\frac{1}{N}\frac{2\sum_{n=1}^{N}y_{n}\bar{y_{n}}}{\sum_{n=1}^{N}y_{n}+\sum_{n=1}^{N}\bar{y_{n}}}$$

(5) $$L_{\mathrm{COMB}}=\alpha L_{\mathrm{DICE}}+\left(1-\alpha \right)L_{\mathrm{BCE}}$$

where $L_{\mathrm{BCE}}$ represents binary cross-entropy and $L_{\mathrm{DICE}}$ represents Dice loss. *N* is the total number of pixels in the image. $y_{n}$ is the predicted softmax pixel value. $\bar{y_{n}}$ is the ground truth of the binary mask.

$$y_{n}\in \left\{0,1\right\}$$

(6) $$\bar{y_{n}}=\left\{\begin{array}{c} 0;\;\mathrm{not}\;a\;\mathrm{road}\;\mathrm{pixel}\\ 1;\;\mathrm{road}\;\mathrm{pixel} \end{array}\right.$$

α=0.75 worked best experimentally. All models are trained using the same cost function.

Along with the loss function, various metrics are tracked during the training. Precision is defined as the percentage of correctly classified road pixels in the predicted road mask, while recall is defined as the percentage of matched road pixels in the ground truth. F1-score is the harmonic mean of precision and recall. IoU is the *intersection-over-union* metric, which measures pixel overlap between predicted road map and ground truth images.

(7) $$\mathrm{Precision}=\frac{\mathrm{TP}}{\mathrm{TP}+\mathrm{FP}}$$

(8) $$\mathrm{Recall}=\frac{\mathrm{TP}}{\mathrm{TP}+\mathrm{FN}}$$

(9) $$F1\text{-}\mathrm{Score}=\frac{2\mathrm{TP}}{2\mathrm{TP}+\mathrm{FN}+\mathrm{FP}}$$

(10) $$\mathrm{IoU}=\frac{\mathrm{TP}}{\mathrm{TP}+\mathrm{FP}+\mathrm{FN}}$$

Here, *TP* denotes true positive, *FP* denotes false positive, and *FN* denotes false negative.

#### 2.2.6. Training Strategy

The U-net++ model was implemented using the Pytorch framework with the help of segmentation models from the PyTorch library (https://github.com/qubvel/segmentation_models.pytorch, accessed on 21 February 2022) version 0.2.0. This paper adopts a two-step training strategy to train the model. All models were trained on Nvidia Tesla GPU P100-PCI-E-16GBx1 (NVIDIA Corporation, Santa Clara, CA, USA).

U-net++ takes 0.22 s for inference whereas U-net takes 0.13 s for inference of a single 256 × 256 image (these results are an average of 100 runs over Nvidia Tesla K80 GPU). Hence, Unet++ might not be suitable for real-time application, but provide significant gains for accurate road area estimation.

#### 2.2.7. First Step

For the Pix2Pix dataset, we use randomly resized crops of size 512 to train the model. This allows us to use a maximum training batch size of 8 on our hardware. For the Massachusetts dataset, where the original images were of sizes 1500, the images are resized to 256 for training, but the inference was performed on the original 1500-sized images padded to 1536. The extensive augmentations as described in the *Augmentations* section are used to provide variations to the dataset. The model is initialized with weights trained on the ImageNet dataset. Adam optimizer is used with a learning rate of 1 ×$10^{-3}$, the model is trained for 20 epochs, and the learning rate is decreased by 0.1 factor on epoch 3, 5, 7, 9, 10, and 12.

#### 2.2.8. Second Step

The model is reinitialized with the best weights from the previous step. The augmentations are decreased to only random cropping, a learning rate of 1 ×$10^{-5}$ for 20 epochs, decreasing every 2 epochs by a 0.1 factor, is used for training.

Finally, the weights with the best validation $L_{\mathrm{COMB}}$ in the second step are loaded and pass the output to the post-processing pipeline.

#### 2.2.9. Post-Processing Pipeline

Segmentation models output the softmax probabilities that range from 0 to 1. They are the probabilities of the pixel being a part of the road. The ground truths in the dataset are the binary images of 0 s and 1 s. To convert the soft probability map into a binary image, we follow an extensive pipeline to clean up the noisy predictions from shadows, buildings, and open grounds, etc. Our image processing operations are inspired by a paper by Adam Van Etten [19] and the solutions to the Spacenet challenge [20].

**Figure 6:**
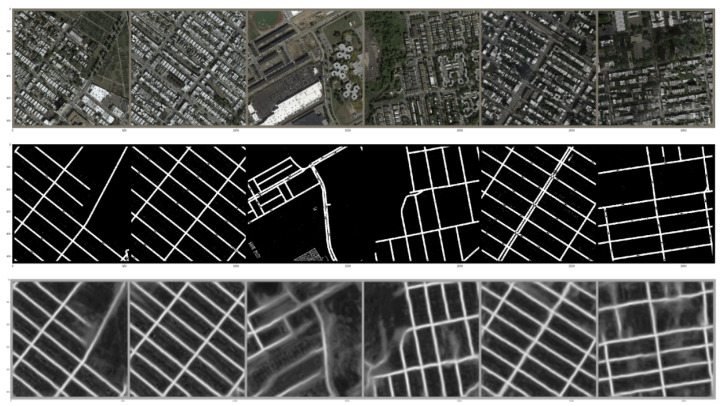
U-net++ with ResNeXt trained on Pix2Pix dataset with 2-stage training strategy. The first row and second row represent the input and ground truth, respectively. The last row represents the model outputs.

In Figure 6, model predictions are visualized. We can see that cleaning up some noise from the images can improve predictions considerably. The pipeline consists of the following stages.

#### 2.2.10. Median Blurring

**Figure 7:**
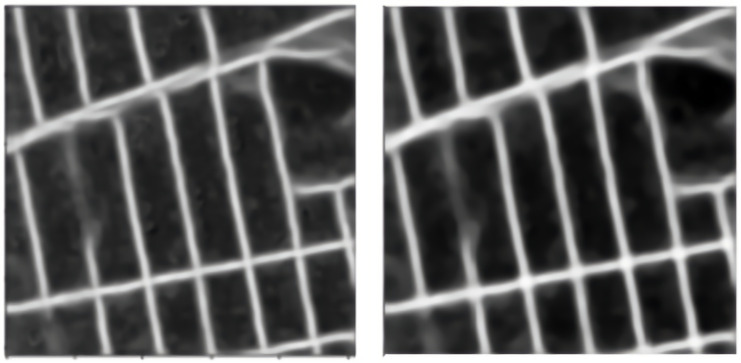
Median blurring operation. The left side is that before applying the filter and the right side is that after applying the filter.

Median blurring is a non-linear filter—median filters. It replaces the pixel values with the median value available in the local neighborhood of the kernel. Median filters are also edge-preserving [21], hence they help us. Median blurring darkens the non-road-map pixels and highlights the roads. We use a square kernel of size 15 × 15 in our experiments. The median blurring on a sample prediction from Pix2Pix is visualized in Figure 7.

#### 2.2.11. Gaussian Adaptive Thresholding

Adaptive thresholding is a technique where a different threshold is calculated for every local region in the image. This technique makes the assumption of an approximately uniform illumination in a smaller image region. The image is divided into sub-images of equal sizes, binarized using a local threshold, and then the results are interpolated to the original image size.

**Figure 8:**
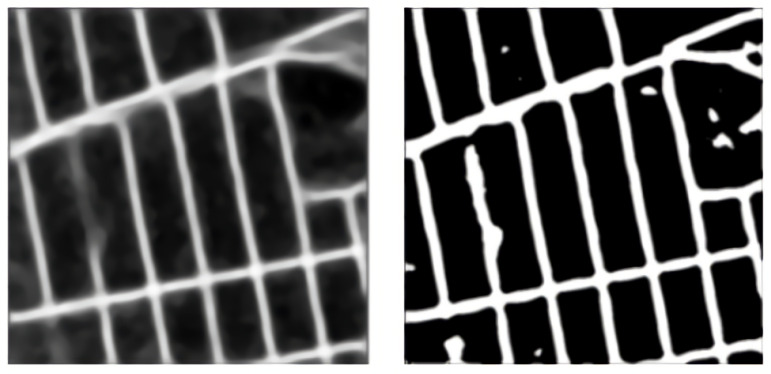
Gaussian adaptive thresholding. The left side is the original image and the right side is the thresholded output.

In adaptive Gaussian thresholding, the threshold value is the weighted sum of neighborhood values, where weights are a Gaussian window. We apply the Gaussian threshold on 85 × 85 patches of the image. The Gaussian adaptive thresholding for the sample Pix2Pix image is shown in Figure 8.

#### 2.2.12. Removal of Small Connected Objects

After adaptive thresholding, there are small blobs that act as noise in the image. There is a need to remove all blobs smaller than a fixed size. The blobs are defined on the basis of connectivity.

“Connectivity” determines which elements of the output array belonging to the structure are considered as neighbors of the central element. Elements up to a squared distance of “connectivity” from the center are considered neighbors. Here, we define connectivity as all the non-diagonal elements as being neighbors.

**Figure 9:**
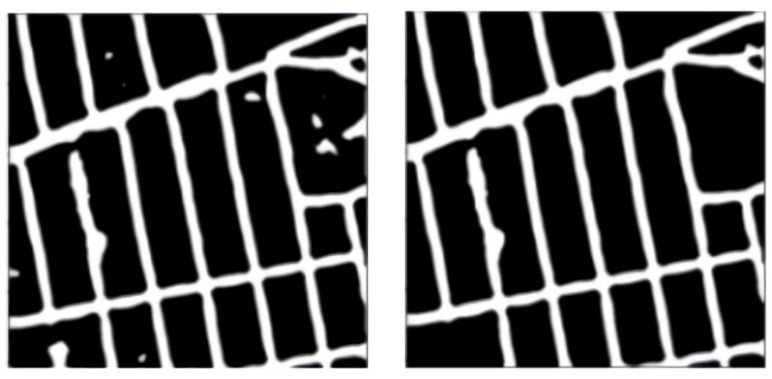
Connected small blob removal. The left side is the original image and blob cleaned output.

In our work, we remove connected objects that are smaller than an empirically selected value of 4900 pixels. The removal of connected blobs is shown in Figure 9.

#### 2.2.13. Small Kernel Erosion

Erosion erodes away the boundaries of the road in the image. We take a small kernel and slide this 2D kernel. A pixel in the original image (either 1 or 0) will be considered 1 only if all the pixels under the kernel are 1, otherwise, it is made 0.

**Figure 10:**
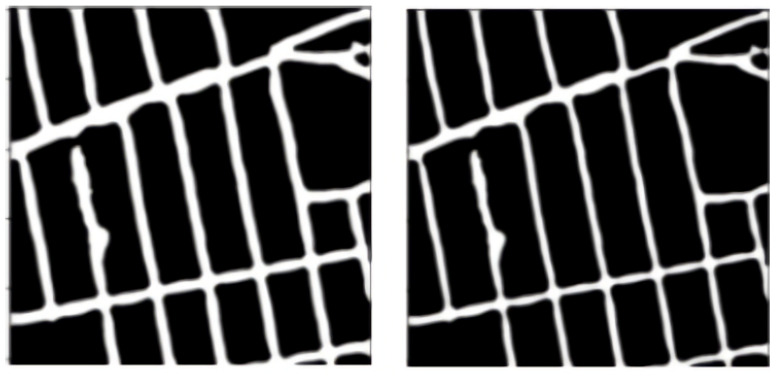
Small kernel erosion. The left side is the uneroded image and the right one is the small kernel-eroded image.

We use a small square kernel of a 3 × 3 size, so as not to break any connection between the roads. This small kernel erosion process is visualized in Figure 10.

## 3. Results

We apply our proposed model and post-processing pipelines to the prepared Pix2Pix dataset. The results are reported in Table 1. We also compare our approach to previous works on the Massachusetts dataset in Table 2.

Here, PP denotes the post-processing pipeline. We find that using post-processing gives us an improvement in terms of Precision, F1-score, Dice score, and IoU. The recall, on the other hand, does not improve.

### 3.1. Recall Study

Recall is a measure of our model correctly identifying true positives. Out of all the correct road pixels, how many of those can our model identify? We see that while cleaning up noisy pixels in pre-processing, some of the correct road pixels are affected too, and this decreases recall slightly; however, it greatly increases precision. The net increase in F1-score in Table 1 tells us that an increase in precision has more effect than a decrease in recall.

**Figure 11:**
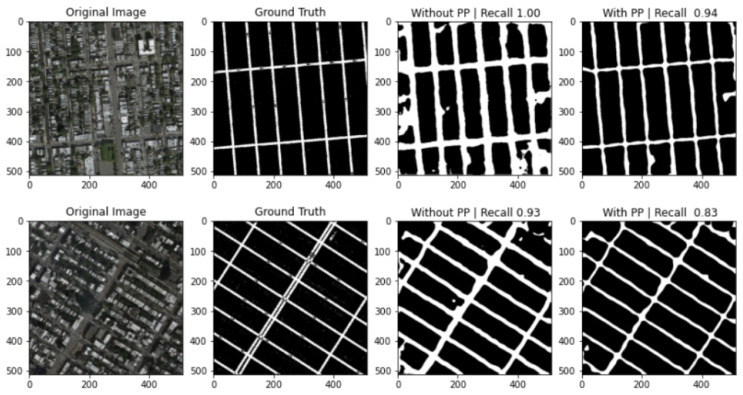
Some example predictions from Pix2Pix datasets with and without post-processing pipeline along with recall scores.

In Figure 11, we can see the examples where post-processing decreased the recall of the model. The post-processing pipeline cleans up the noise in the image and makes the road network better. This is important, because for the process of road area calculation we need to clean up as much noise as possible.

### 3.2. Area Calculation

In the previous sections, we saw an improved deep learning model for roadway extraction. The next step in our two-step process for area calculation is *pixel-area estimation*.

The resolution of the images is then considered, which is around 0.5 m/pixel for Pix2Pix and 1 m/pixel for the Massachusetts dataset. With the help of this resolution, we calculate the total road area by multiplying it with the road pixel area.

(11) $$R_{area}=I_{res}\times \sum_{i=1}^{S}\sum_{j=1}^{S}R_{mask_{i,j}}$$

Here, $I_{res}=0.5\times 0.5$ = 0.25 m${}^{2}$/$px^{2}$. $R_{mask_{i,j}}$ is a binary road mask where 1 denotes road and 0 denotes non-road area. Taking the sum gives us the total road pixel area.

**Figure 12:**
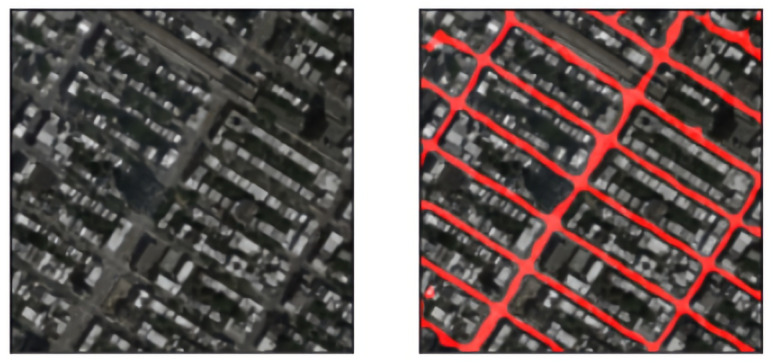
A sample prediction on Pix2Pix using the trained model, which is post-processed and overlaid on the original image.

In Figure 12, we can see the road segment in the red color. The total number of red pixels is the road pixel area in the image. The sum comes out to be 48,498 pixels, hence the road area calculated will be 48,498 $px^{2}$× 0.25 m${}^{2}$/$px^{2}$ = 12,124.16 m${}^{2}$.

**Figure 13:**
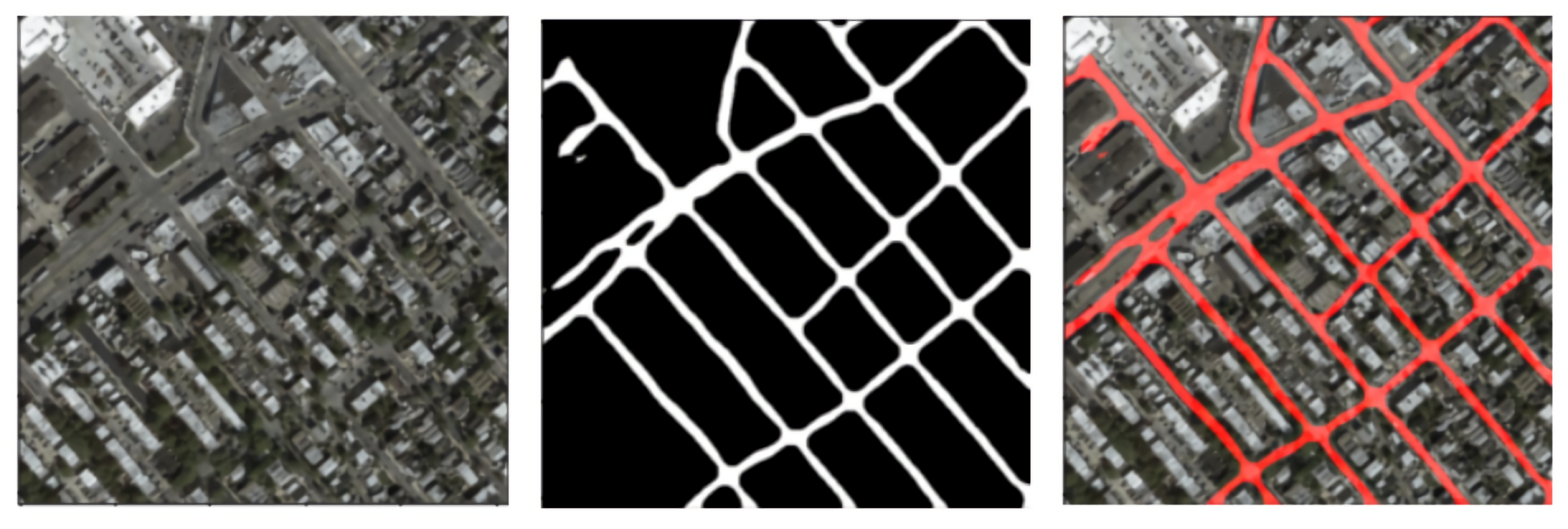
Another sample prediction on Pix2Pix using the trained model, which is post-processed and overlaid on the original image.(Left to Right) Satellite image, road mask, overlaid road mask.

Another example can be seen in Figure 13. Here, the road pixel area is 42,453 $px^{2}$, hence the road area calculated will be 42,453 $px^{2}$× 0.25 m${}^{2}$/$px^{2}$ = 10,613.5 m${}^{2}$.

**Figure 14:**
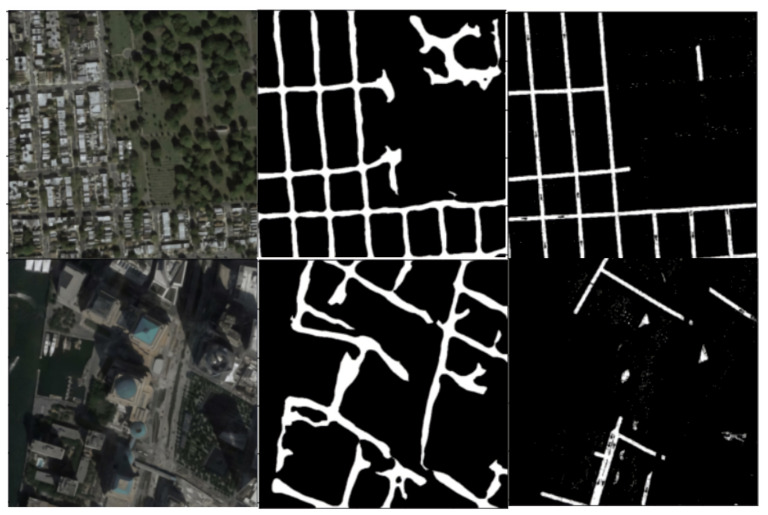
Examples of misclassifications (left to right in each row): satellite image, post-processed output, ground truth image.

**Figure 15:**
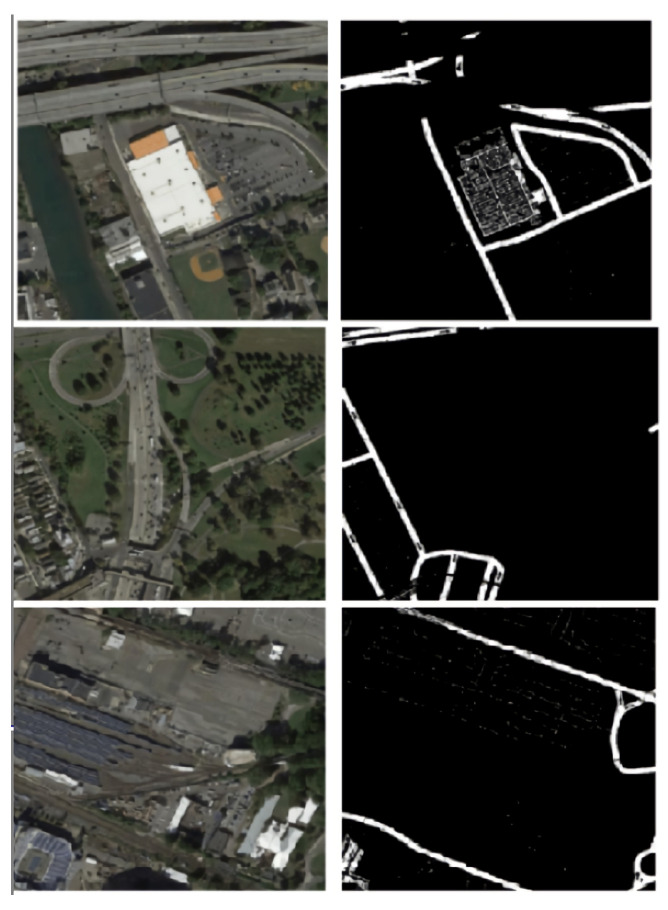
Some noisy labels in the Pix2Pix dataset: the crude method of creating labels by applying thresholds on the black and white image introduced errors into the dataset, thereby limiting the performance of models.

Area calculation is highly dependent on the segmentation accuracy of road pixels from the satellite image. Misclassification errors in the road pixel mask can result in a deviation of the estimated area from the actual value. Figure 14 presents some erroneous predictions from the model. This can be attributed to two reasons: (a) Effects of external factors such as lakes, rivers, trees, and buildings along with their shadows occlude the roadway area and decrease interpretability. (b) Noisy labels in the dataset limit the model’s performance (examples in Figure 15).

## 4. Related Work

In the previous few years, significant attention has been received and extensive research solutions have been proposed to tackle the problem of roadway extraction. Initial approaches included skeleton-based, road-line extraction techniques, such as in references [24,25]. The road centerline can also be obtained from image processing operations, such as morphological thinning algorithms [26].

Road extraction from openly available satellite images can be considered as an image segmentation or pixel-level classification problem. For example, Song and Mingjun [27] use pixel spectral information and image-segmentation-derived object features for road extraction. Alshehii and Marpu [23] use morphological filtering followed by graph-based segmentation for extracting road networks from high-resolution satellite images.

These described research studies are mainly obtained by data-driven and heuristic methods. The basis of these preliminary studies is unsupervised learning, such as global optimization and graph methods dependent on color features. A drawback of using color features is color sensitivity. If the obtained road maps are of different colors, the unsupervised algorithms will perform poorly. Hence, to accurately extract road networks of various scales from remote-sensing satellite images, new robust methodologies, such as deep learning algorithms, are required.

Recent years have seen great strides in the field of deep learning. Deep neural networks now provide state-of-the-art solutions to many computer vision tasks. Image classification [28], scene recognition [29], and object detection [30] all have deep convolutional networks as their fundamental building blocks.

The field of remote sensing has also seen a rise of deep neural networks in its study. Reference [18] uses synthetic road labels to train neural networks to learn features and generate predictions with broad context. References [22,31] perform object detection and extraction with convolutional neural networks (CNN). These studies outperform the traditional road extraction methods.

Zhong et al. [1] proposed a CNN model and estimated the influence of filter stride, learning rate, input data size, training epoch, and fine-tuning for building and road extraction. They use the Massachusetts Roads dataset for this purpose. Wei et al. [2] proposed a refined CNN for road extraction in aerial images. It consisted of both deconvolutional and fusion layers. Reference [23] used a patch-based CNN for extracting roads and buildings simultaneously. Panboonyuen et al. [3] presented an encoder–decoder deconvolution network using SegNet [4] trained using ELU [5] with eight-times data augmentation.

Zhang et al. [6] proposed a deep residual U-net for road semantic segmentation from high-resolution satellite imagery. The proposed network included residual connections, which allowed fewer parameters and facilitated information propagation to counter the vanishing gradient problem [7]. It was trained and tested on the Massachusetts dataset to show improved results. Many new approaches for road-space extraction were proposed using U-net-based architectures. Reference [8] describes road extraction from high-resolution spatial remote-sensing images using richer convolutional features. Reference [9] describes the dense U-net architecture while reference [10] describes the Y-net architecture. Cascaded end-to-end convolutional neural networks were introduced in reference [11]. References [12,13] use U-net models for road-space extraction.

## 5. Conclusions

This paper proposed the extraction and calculation of the roadway area from satellite images using an improved version of U-net and ResNet—U-net++ and ResNeXt—for state-of-the art performance. An extensive training strategy was performed with a hybrid loss, and augmentations were introduced to add variations to the dataset. The proposed model was trained on the modified Pix2Pix dataset and Massachusetts dataset. Results were compared on the Massachusetts dataset with the previous models trained on the same, and it is shown that the proposed model improves the existing U-net F1-score baseline by 3%.

This paper also provided a strategy to calculate the area of road covered in the satellite image using pixel-area estimation. The proposed approach made it possible to calculate the roadway area of any satellite image. This framework can be applied to any openly available satellite images to estimate the road area.

The Pix2Pix dataset used to train the model was generated by code and hence introduced noise. High-resolution satellite imagery with road areas labeled should be worked upon for an accurate model.

Various deep learning approaches were proposed after ResNet (apart from ResNeXt), such as Efficientnet [32], NFNet [33], and novel transformer architectures [34]. Using these models as a backbone for U-net or U-net++ is a research area that needs to be further explored. More segmentation models other than U-net family-like RCNN [35] and GAN-based approaches [36] remain to be applied on the Pix2Pix dataset. The Pix2Pix dataset used Google Images for its source. Further work on extending the area calculation approach to any random sample of images can be performed to improve accessibility to the experts working in the field of remote sensing.

## Figures and Tables

**Table 1:** Results on Pix2Pix validation dataset with and without post-processing.

Metric	Without PP	With PP
Precision	0.41	0.54
Recall	0.86	0.76
F1-score	0.27	0.31
Dice score	0.54	0.62
IoU	0.39	0.47

**Table 2:** Comparison of the model with previous approaches on Massachusetts Test Roads dataset: metrics that were not available for all the papers were excluded.

Model	Precision	Recall	F1score
Panboonyuen et al. [20]	0.858	0.894	0.876
Mnih-CNN [18]	0.887	0.887	0.887
Mnih-CNN + CRF [18]	0.890	0.890	0.890
Mnih-CNN + Post-Processing [18]	0.901	0.901	0.901
Saito-CNN [22]	0.905	0.905	0.905
U-net [14]	0.905	0.905	0.905
Alshehii et al. [23]	0.917	0.917	0.917
ResU-net [6]	0.919	0.919	0.919
U-net++ + ResNeXt	0.943	0.951	0.947

## Data Availability

Not applicable.
